# Data on interaction between adeno-associated virus and U87 cell via cRGD chemical modification

**DOI:** 10.1016/j.dib.2016.02.009

**Published:** 2016-02-10

**Authors:** Chuanling Zhang, Tianzhuo Yao, Yongxiang Zheng, Zhongjun Li, Lihe Zhang, Demin Zhou

**Affiliations:** State Key Laboratory of Natural and Biomimetic Drugs, School of Pharmaceutical Sciences, Peking University, No. 38, Xueyuan Road, Beijing 100191, China

**Keywords:** Adeno-associated virus, Viral modification, Targeted gene delivery

## Abstract

RGD tripeptide is a specific, high-affinity ligand for integrin, which is highly expressed in cancer cells. We previously reported that cRGD chemically modified AAV2 (AAV2^N587+1/azido+RGD^) showed significantly enhanced infectivity compared to RGD genetically inserted AAV2 (AAV2^N587+RGD^) (10.1016/j.biomaterials.2015.11.066) [1]. Herein we provide the binding ability analysis of RGD modified AAV2 and U87 cell by flow cytometry and the theoretical working model of RGD–αvβ3 integrin interaction.

## 1 **Specifications table**

**Table:** 

Subject area	*Biology, Chemistry*
More specific subject area	*Gene therapy*
Type of data	*Figure*
How data was acquired	*Flow cytometry*
Data format	*Analyzed*
Experimental factors	*Site-specific modification of AAV2 with cRGD*
Experimental features	*Binding ability between AAV2 and U87 was analyzed by Flow Cytometry*
Data source location	*Peking University, Beijing, China*
Data accessibility	*Data is within this article and at Protein data bank PDB: 1L5G, PDB: 1LP3*

## 2 Value of the data

• This data set will be of value to the scientific community wanting to analyze the binding ability of virus and host cell.
• The data show new way to study the biological mechanisms of AAV2 entry.
• The data may stimulate further research on viral targeted gene delivery.

## 3 **Data**

The data shared in this article is the experimental and theoretical analysis of interaction between cRGD modified AAV2 and host cell (U87).

## 4 Experimental design, materials and methods

### 4.1 Cell lines

U87 cells were maintained in an atmosphere containing 5% CO_2_ in Dulbecco’s modified Eagle’s medium (Gibco, Carlsbad, CA, USA) supplemented with 10% fetal bovine serum (FBS; PAA, Austria) and 2 mM l-glutamine (Gibco).

### 4.2 Cell surface binding assays

**Fig. 1:**
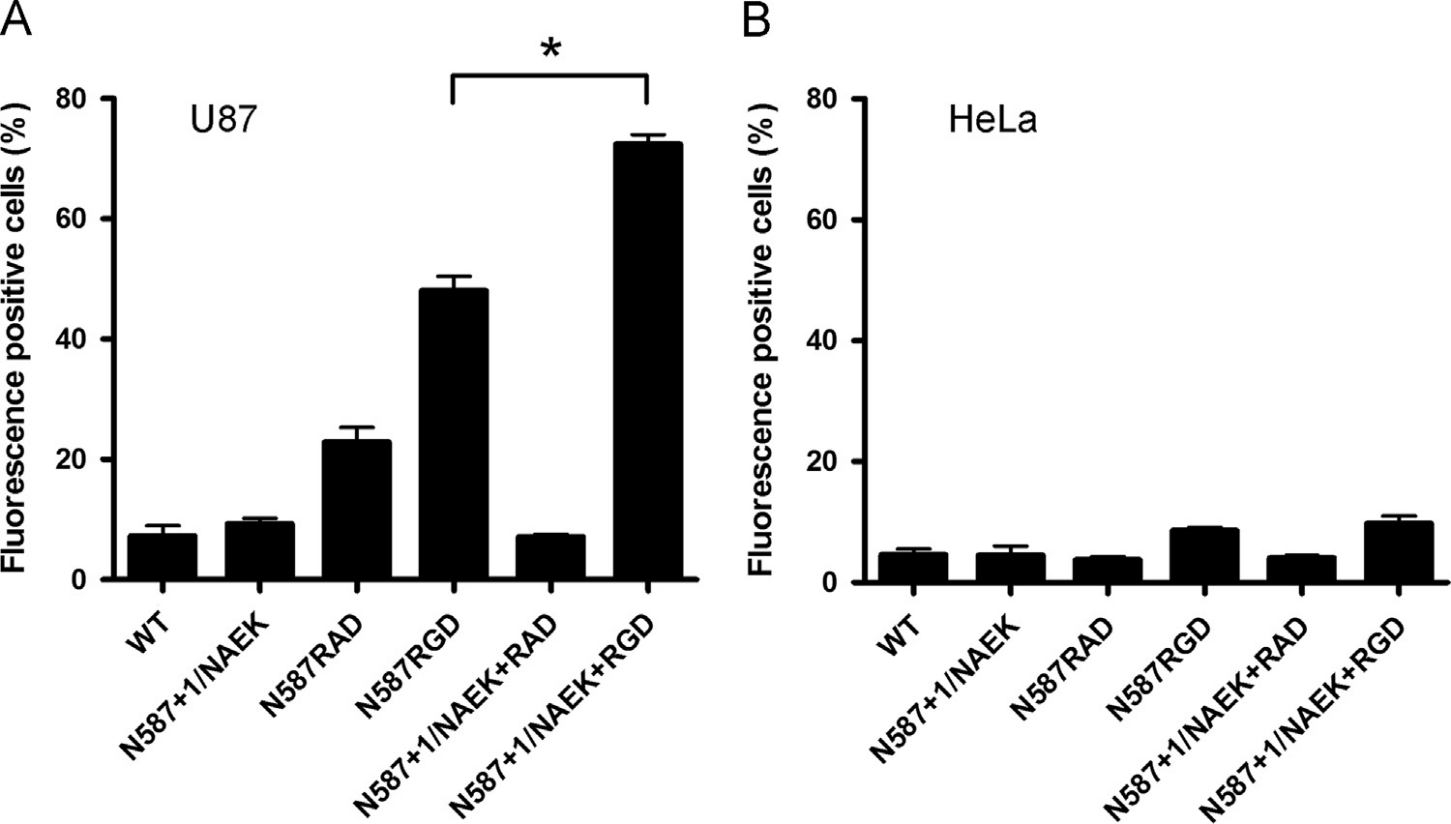
Analysis of the binding ability of vector particles with HeLa and U87 cells.

**Fig. 2:**
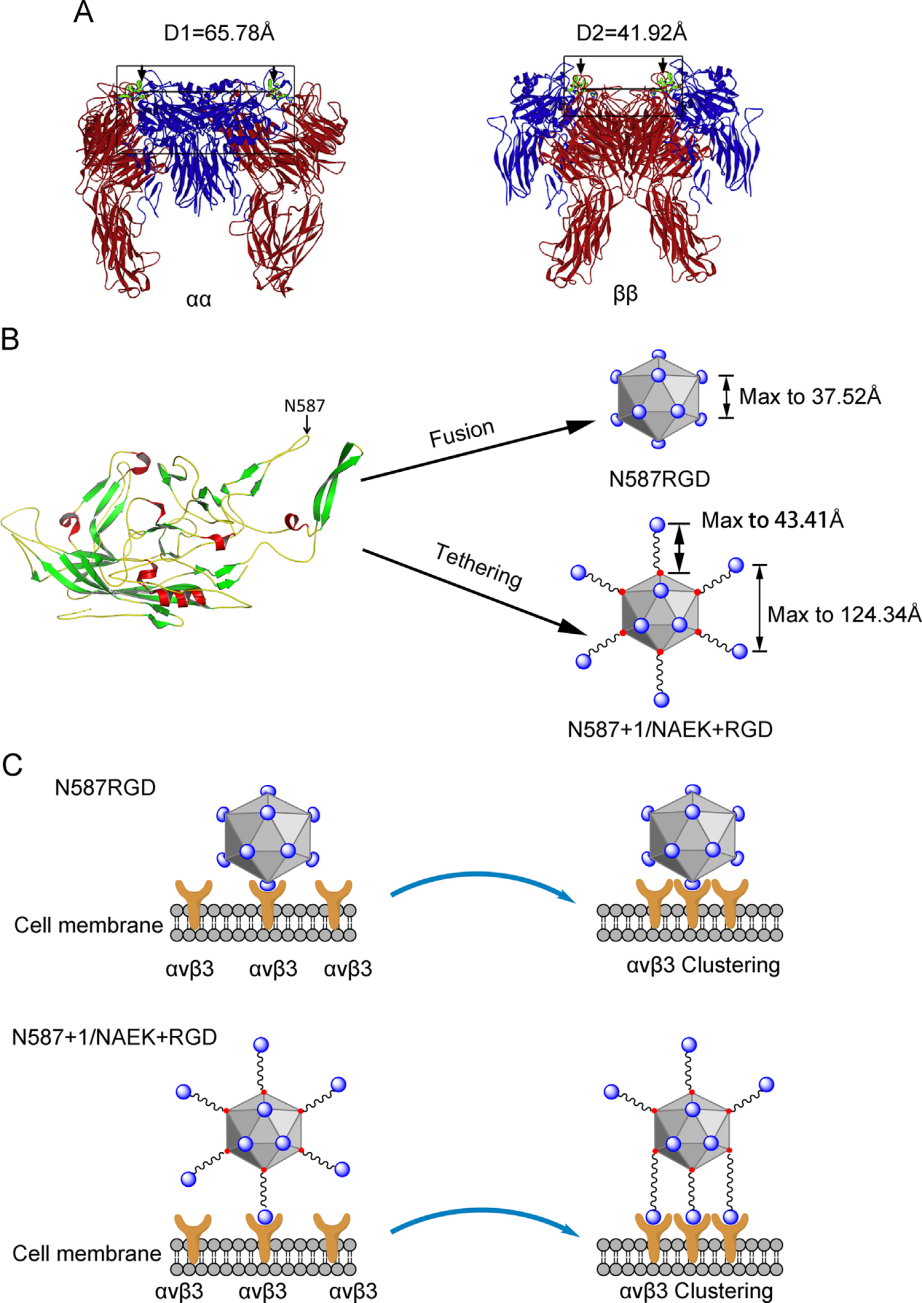
Theoretical analysis of the different effects of RGD tethering versus RGD fusion on improvement of tropism selectivity [2], [3], [4]. (A) The three-dimensional model of αvβ3 receptor clustering. The distance between clustering αvβ3 molecules for RGD binding was labeled accordingly. Black arrows indicate RGD binding sites. (B) Schematic representative of the structure of RGD tethering versus RGD fusion to the AAV capsid protein at site N587+1. The distance between the two adjacent sites of RGD fused on AAV2 was 37.52 Å. The length of DIBO-cRGD was 43.41 Å. Upon tethering of cRGD via a DIBO linker, the maximum distance between two cRGD on AAV2^N587+1/NAEK+RGD^ increased to 124.34 Å (2×43.41 Å+37.52 Å=124.34 Å). (C) Schematic illustration of the interactions between the clustering αvβ3 receptor and adjacent RGD-tethered versus RGD-fused ligands within the AAV2 vector. The distance between two adjacent RGD fusion motifs (~37.52 Å) was much shorter than the distance between the clustering αvβ3 binding sites (either 65.78 or 41.92 Å), preventing simultaneous binding. In contrast, the distance between the two adjacent tethered RGD motifs on AAV2^N587+1/NAEK+RGD^ was 124.34 Å, allowing simultaneous binding of multiple integrin αvβ3 receptors. Blue indicates the RGD motifs. (For interpretation of the references to color in this figure legend, the reader is referred to the web version of this article.)

Cells were resuspended at a density of 2×10^6^ cells/mL in binding buffer containing 5% FBS. Equal amounts of viral vectors were incubated with cells at 4 °C for 2 h, and unbound vector particles were then removed by washing with PBS. Vector particles bound to HeLa or U87 cells were detected by staining with anti-AAV A20 monoclonal antibodies and subsequent FACS analysis. *P<0.05 versus the corresponding control (Fig. 1, Fig. 2).
